# Genetic Relatedness among Hepatitis A Virus Strains Associated with Food-Borne Outbreaks

**DOI:** 10.1371/journal.pone.0074546

**Published:** 2013-11-06

**Authors:** Gilberto Vaughan, Guoliang Xia, Joseph C. Forbi, Michael A. Purdy, Lívia Maria Gonçalves Rossi, Philip R. Spradling, Yury E. Khudyakov

**Affiliations:** Division of Viral Hepatitis, Centers for Diseases Control and Prevention, Atlanta, Georgia, United States of America; Institut Pasteur, France

## Abstract

The genetic characterization of hepatitis A virus (HAV) strains is commonly accomplished by sequencing subgenomic regions, such as the VP1/P2B junction. HAV genome is not extensively variable, thus presenting opportunity for sharing sequences of subgenomic regions among genetically unrelated isolates. The degree of misrepresentation of phylogenetic relationships by subgenomic regions is especially important for tracking transmissions. Here, we analyzed whole-genome (WG) sequences of 101 HAV strains identified from 4 major multi-state, food-borne outbreaks of hepatitis A in the Unites States and from 14 non-outbreak-related HAV strains that shared identical VP1/P2B sequences with the outbreak strains. Although HAV strains with an identical VP1/P2B sequence were specific to each outbreak, WG were different, with genetic diversity reaching 0.31% (mean 0.09%). Evaluation of different subgenomic regions did not identify any other section of the HAV genome that could accurately represent phylogenetic relationships observed using WG sequences. The identification of 2–3 dominant HAV strains in 3 out of 4 outbreaks indicates contamination of the implicated food items with a heterogeneous HAV population. However, analysis of intra-host HAV variants from eight patients involved in one outbreak showed that only a single sequence variant established infection in each patient. Four non-outbreak strains were found closely related to strains from 2 outbreaks, whereas ten were genetically different from the outbreak strains. Thus, accurate tracking of HAV strains can be accomplished using HAV WG sequences, while short subgenomic regions are useful for identification of transmissions only among cases with known epidemiological association.

## Introduction

Hepatitis A virus (HAV), the causative agent of hepatitis A, is responsible for approximately 1.5 million reported cases and tens of millions of infections each year, accounting for most acute viral hepatitis worldwide [1]. Although hepatitis A is a self-limited liver disease [2], it occasionally progresses to severe, life-threatening illness [3], [4].

HAV is a single-stranded, positive-sense RNA virus belonging to family *Picornaviridae* [2]. The HAV genome is approximately 7,500 nucleotides (nts) in length, consisting of a 5′ untranslated region (UTR) of ∼740 nts, a coding region of ∼6,681 nts and a 3′ UTR of 40–80 nts [5]. The coding region encodes a large polyprotein, which was divided into segments P1, P2 and P3. P1 is processed into three major proteins of the viral capsid, VP1, VP2 and VP3, and VP4, which plays a role in virion assembly [5]. Nonstructural proteins are generated by protease cleavage of P2 and P3 [5]. The genome is heterogeneous, allowing classification of HAV into six genotypes and several subgenotypes, [6], [7], [8], [9]. Heterogeneity has a limited effect on antigenic diversity and results in the existence of a single serotype [10]. HAV genotypes and subtypes are not uniformly distributed across the world. Subtypes IA and IB are prevalent in North and South America, Europe, China, Japan and Thailand [11]. The majority of human non-genotype I strains belong to subgenotype IIIA and circulate in India, Kazakhstan, Europe and the United States [12], [13]. The most prevalent subgenotype in the United States is IA, followed by subgenotypes IB and IIIA [8], [12].

The prevalence of HAV infection varies in different regions of the world [12]. In low prevalence countries, outbreaks of hepatitis A have a significant impact on public health [14]. In the United States, large multi-state outbreaks of hepatitis A associated with contaminated food have been reported [15], [16], [17]. In 2003, 1,023 cases of hepatitis A were reported in Tennessee, North Carolina, Georgia and Pennsylvania, associated with consumption of green onions [16], [17]. In 2005, 39 cases of hepatitis A were reported in Alabama, Florida, South Carolina and Tennessee among individuals who had consumed oysters [15]. Molecular analysis of the VP1/P2B region showed that sequences of HAV strains associated with contaminated onions could be classified into 3 different genetic groups, and sequences of HAV associated with consumption of oysters belonged to a different genetic group. All four groups belonged to subgenotype IA. Each group had identical VP1/P2B sequences [16], [17], [18].

The genetic diversity of HAV is usually evaluated using different subgenomic regions [7], [9], [19]. Although sequences of the VP1/P2B junction are most frequently used to identify genotypes, track transmission and assess phylogenetic relationships among HAV strains [8], [15], [16], [17], little is known about how accurately this region or other subgenomic regions represents HAV genetic heterogeneity.

This molecular analysis details the genetic heterogeneity among HAV variants identified during the aforementioned multi-state, food-borne outbreaks.

## Materials and Methods

### Serum specimens

Serum specimens (n = 101) from four large food-borne outbreaks [15], [16], [17], were randomly selected for WG sequencing. Specimens were collected from: three outbreaks in 2003 associated with consumption of green onions in Tennessee (outbreak A; n = 18), Pennsylvania, Ohio and West Virginia (outbreak B; n = 34), North Carolina and Georgia (outbreak C; n = 25) [16], [17] and a multistate outbreak in 2005 associated with consumption of oysters (outbreak D; n = 24) [18]. An additional sample set (n = 14) derived from sporadic (i.e., non-outbreak) strains sharing VP1/P2B sequences with strains from outbreaks A, B and D was obtained. The specimens had been collected between 1998 and 2008 from the Sentinel Counties Surveillance for Acute Viral Hepatitis and Border Infectious Disease Surveillance Projects [8].

### Full-length HAV genome amplification and sequencing

WG sequences were obtained from 16 overlapping DNA fragments amplified using a nested PCR laboratory protocol. Briefly, total RNA was extracted from serum samples using the automated MagNA Pure LC system (Roche Applied Science, Indianapolis, IN) and Total Nucleic Acid Isolation kit (Roche). The extracted material was then subjected to reverse transcriptase (RT)-PCR using the one-step RT-PCR kit (QIAGEN, Valencia, CA) to obtain 16 amplicons encompassing the entire length of the HAV genome. The amplicons were then used as templates for second-round PCR using SYBR Green Perfecta kit (Quanta BioSciences, Gaithersburg, MD). PCR primers sequences are shown in Table 1. PCR amplification was carried out on LightCycler 480 (Roche). Melting curve analysis was performed to identify PCR-positive samples. Both DNA strands were sequenced using the automated 3100 genetic analyzer (Applied BioSystems, Foster City, CA). WG sequences reported in this work have been deposited in GenBank.

**Table 1:** Primers sequences for WG amplification.

RT Primers			Nested Primers		
Set	Primer ID	Sequence	Set	Primer ID	Sequence
1	EFWDHA0008	CACCGCCGTTTGCCTAGGC	1	IFWDHA012	CGCCGTTTGCCTAGGCTATAGGC
	ERVSHA0660	ATGAGGAAAAACCTAAATGCCCCTG		ERVSHA652	AACCTAAATGCCCCTGAGTACCTCAG
2	EFWDHA0418	TTAAGACAAAAACCATTCAACGCCG	2	IFWDHA251	GCTCTCATCCAGTGGATGCATTGAG
	ERVSHA1138	TGGTCACCAGGAACCATGGCAC		ERVSHA1095	GGTGTGGGGTTTATCTGAACTTGAATC
3	EFWDHA0844	GTTGGCTCACATCAAATTGAACCTTTG	3	IFWDHA0865	CCTTTGAAAACCTCTGTTGATAAACCTGG
	ERVSHA1592	GCAGCTAAGGTTGGAATGGATGTCC		ERVSHA1562	GTAAAATGAGTAATTTTAATTCCACCACCTTG
4	EFWDHA1288	ACAATCAGAGTTTGGTCAGAGTTGAATATTG	4	IFWDHA1297	GTTTGGTCAGAGTTGAATATTGGAACAGG
	ERVSHA2095	AATTGCTGAAAGATAAACATTAACTCTAACATGAG		ERVSHA2068	AACATGAGAAGCAACATTAGAAGGAGAAGTC
5	EFWDHA1766	AGGTTTTTCCAACCAAATATCATTCAGG	5	IFWDHA1808	GCTTTGTTCCTGGGAATGAGTTAATAGATGT
	ERVSHA2490	CGAAGACAGAGTTATTGGAAATGTGTACTCTTTATT		ERVSHA2440	TAAAAGTACACAGAAAATGAGACCTTCCCAT
6	EFWDHA2277	GAGTGCAAGCACCTGTGGGAGC	6	IFWDHA2285	CACCTGTGGGAGCTATTACAACAATTGA
	ERVSHA2978	CTACTCATCATGGACTCAGTGGACAACAT		ERVSHA2890	AAATAACAACTAAAGGACAAATATTCATCAGAATG
7	EFWDHA2683	GATTATAAAACTGCCCTTGGAGCTGTTAGATT	7	IFWDHA2696	CCCTTGGAGCTGTTAGATTTAATACAAGAAGAAC
	ERVSHA3390	TTATCATCCTTCATTTCTGTCCATTTCTCATC		ERVSHA3378	CATTTCTGTCCATTTCTCATCATTCAATCT
8	EFWDHA3094	GGGAAACAAAGACTCAAATATGCTCAGG	8	IFWDHA3096	GAAACAAAGACTCAAATATGCTCAGGAAGA
	ERVSHA3848	TAACTGAGCAGCCAATATCTGCATAATTCAT		ERVSHA3827	CATAATTCATAACTCTCAACAAACCAATTATGTG
9	EFWDHA3566	GCAGATAGAATGCTTGGATTGTCTGGAGT	9	IFWDHA3608	AACAGGGTGTTGGATTGATAGCAGAGTG
	ERVSHA4318	CTGATTTATAGATCCAAGATTCTTGAGCTTTTG		ERVSHA4274	CTATGCAATCTCTAAGAGGTGACAAATGAAC
10	EFWDHA4005	ATTGGCTATATACAAAATTGAAGGATTTTTATGA	10	IFWDHA4022	GAAGGATTTTTATGAAGTAAATTATGGCAAGAA
	ERVSHA4739	CAGGTTTAACTTCAACCTTAAAATGAAGCCT		ERVSHA4723	CTTAAAATGAAGCCTACGATCAATTGCTTC
11	EFWDHA4522	GCCAAAATACAACAGATGAAGATTGGTCAG	11	IFWDHA4546	GGTCAGATTTTTGTCAATTAGTGTCAGGATG
	ERVSHA5180	GTCACGCCATGATAAACCCCTTCAG		ERVSHA5170	ATAAACCCCTTCAGCTGGAATTGGTTC
12	EFWDHA4880	GATGACAGTTGAAATTAGGAAACAAAATATGAGTG	12	IFWDHA4905	AAAATATGAGTGAATTCATGGAGTTGTGGTC
	ERVSHA5703	TCTACAGTCAAATCAACTGTAGTACCATCATTCTT		ERVSHA5630	GTCCCTCAGAAATTAACATAGGAGTTCCATT
13	EFWDHA5334	GGATGATTGGTTGTTAGTACCTTCTCATGC	13	IFWDHA5362	CTTATAAATTTGAAAAGGATTATGAAATGATGGA
	ERVSHA6108	AAAAACTGAAGCTTCTTTGTAATCCTCTGG		ERVSHA6086	CCTCTGGTTCTTCCACAATAGGTAATGAATAT
14	EFWDHA5735	CCTGGAATGTGTGGTGGGGC	14	IFWDHA5785	GCAATTTTGGGTATTCATGTTGCTGG
	ERVSHA6580	TTAAATAATTCATCCCACTGTCTATCAGGATC		ERVSHA6560	GTCTATCAGGATCTATGCCAATAGCAACAC
15	EFWDHA6252	AAGAGATTTAATTTGGTTGGATGAAAATGGT	15	IFWDHA6286	CTGCTAGGAGTTCATCCAAGATTGGC
	ERVSHA7002	TTGAACTCATCCACAATTTTCTGTCCAAT		ERVSHA6980	TCCAATCAAATCAAGATTATCAATTTGAACATC
16	EFWDHA6668	GGTAGAATCATGAGTGAATTATCTGGAACACC	16	IFWDHA6676	CATGAGTGAATTATCTGGAACACCATCTCA
	ERVSHA7370	TTGTTTAAACAAATCATGAAAGGTCACAAATG		ERVSHA7356	CATGAAAGGTCACAAATGAAACACTGGTC

### Sequence analysis

The electrophoregrams were analyzed and sequence contigs were built and edited to reconstruct the HAV WG using SeqMan v8.1, Lasergene Package, (DNAstar Inc., Madison, WI). The Kalign algorithm (http://www.ebi.ac.uk/Tools/kalign) was used to create alignments and analyze sequences. The alignment was inspected and optimized manually. Phylogenetic and molecular evolutionary analyses were conducted using MEGA version 4 [20] and the Wisconsin Sequence Analysis Package (Genetic Computer, Madison, WI) as described elsewhere [16]. Median joining networks (MJN) were constructed using DNA Alignment (http://www.fluxus-technology.com/) and Network software v4.5.1.6 [21].

### Nucleotide diversity analysis

The average number of nt differences *per* site between two sequences, nt diversity (π), were computed using DnaSP v5.1 [22]. A sliding window method, with windows of 500 nts and steps of 25 nts, was used to calculate π across the entire alignment for each independent outbreak. Values for each window were calculated and assigned to the nt position at the midpoint of each overlapping window.

### Phylogenetic tree distance analysis

Independent alignments for each outbreak were scanned using windows of 500 nt and steps of 25 nts. Thus, 1370 different independent alignments encompassing the full-length viral genome for each outbreak were generated. The genetic distances (Kimura 2-parameter) were calculated using DNAdist as implemented in Phylip v3.5. Subsequently, the corresponding Neighbor-Joining phylogenetic trees for each matrix were constructed as described elsewhere [23]. The resulting trees were compared to the WG tree using the symmetric distance method implemented in the Treedist program. The distances between trees were plotted versus nt positions along the entire genome.

### Intra-host HAV genetic variation

Intra-host HAV genetic variation was assessed using a modified version of end-point limiting-dilution (EPLD) real-time PCR [24], [25]. Briefly, purified RNA was reverse-transcribed into cDNA and serially diluted in quadruplicates (0.25 log dilutions). All dilutions were amplified by two rounds of real-time PCR. The dilution resulting in 50% positivity was considered the end point. Under these conditions approximately 50% of reactions do not carry template molecules and do not generate PCR amplicons. Thus, the amplicons generated are likely to originate from a single template. Multiple PCR clones (30–40 per sample) *per* patient were sequenced.

### Characteristics of individuals with sporadic HAV infection

Epidemiologic data obtained via enhanced viral hepatitis surveillance for the 14 sporadic isolates were reviewed to ascertain whether there were previously unrecognized linkages to the four outbreaks. Case data for sporadic and outbreak isolates were compared in relation to date of symptom onset, geographic location, and risk factors or exposures.

## Results

### Genetic relatedness among HAV variants

**Figure 1:**
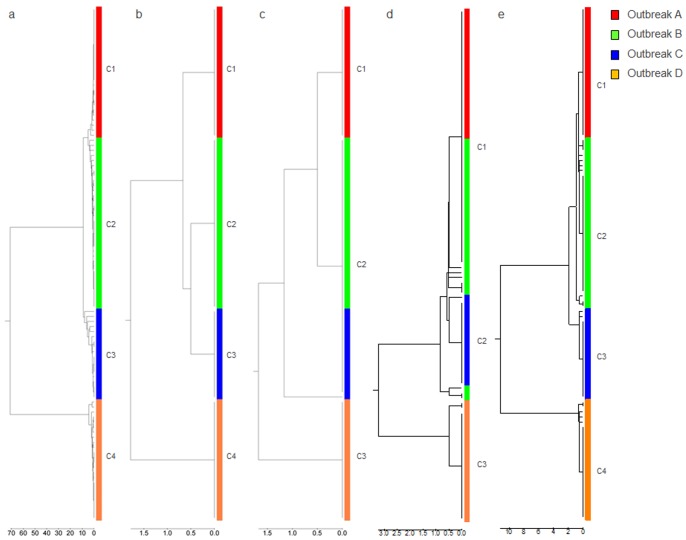
Phylogenetic analysis using different subgenomic regions. Phylogenetic analysis using sequences of (a) WG, (b) VP1/P2B, (c) 5′-UTR, (d) N-terminus of the VP1 gene and (e) entire VP1.

Sixteen overlapping PCR fragments encompassing the entire length of the HAV genome were consistently amplified from all HAV PCR-positive specimens, indicating the uniform sensitivity of all PCR primer sets. Using this protocol, 101 WG sequences were obtained. Phylogenetic analysis supported clustering of the HAV isolates into four groups within subtype IA (Figure 1A). In contrast to VP1/P2B sequences, which were identical within each group, the WG sequences were heterogeneous (Table 2). The WG sequences were less diverse (mean genetic distance, 0. 21; range 0.14–0.27) in groups A, B and C than in group D (mean genetic distance, 1.94; range 1.92–2.03).

**Table 2:** Nucleotide distances among HAV full-length genome outbreak strains.

Group	Outbreak		Minimum (%)	Maximum (%)	Median (%)	Mean (%)	SD
Inter-Outbreak	A	C	0.20	0.44	0.25	0.27	0.06
Inter-Outbreak	A	D	0.08	0.22	0.14	0.14	0.02
Inter-Outbreak	B	C	0.16	0.41	0.20	0.23	0.06
Inter-Outbreak	D	A	1.84	1.99	1.92	1.92	0.03
Inter-Outbreak	D	B	1.80	2.00	1.91	1.90	0.03
Inter-Outbreak	D	C	1.91	2.19	2.02	2.03	0.06
Intra-Outbreak	A	A	0.00	0.11	0.01	0.02	0.02
Intra-Outbreak	B	B	0.00	0.15	0.03	0.04	0.03
Intra-Outbreak	C	C	0.00	0.31	0.05	0.09	0.08
Intra-Outbreak	D	D	0.00	0.15	0.03	0.04	0.04

**Figure 2:**
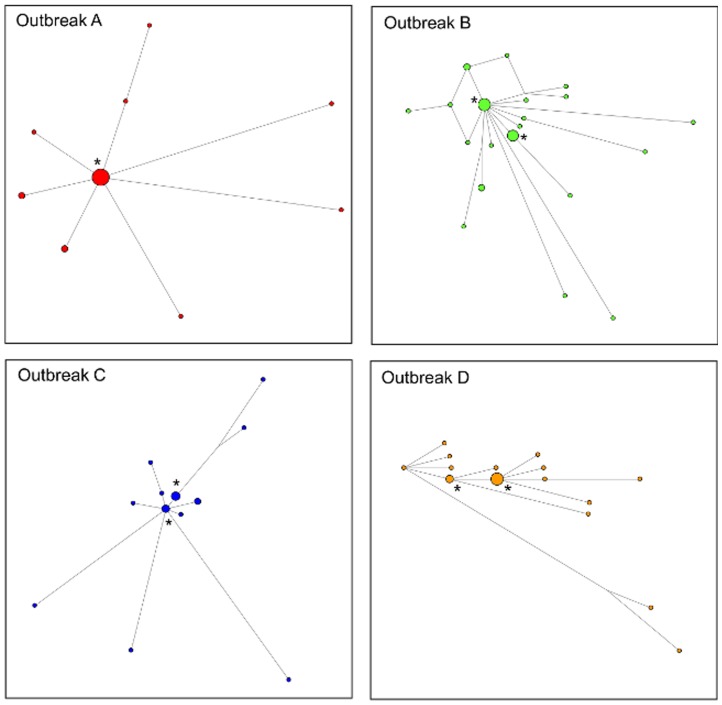
Median joining network analysis. Median Joining Networks constructed using WG sequences. Viral strains from each outbreak are color coded. Major HAV variants are denoted by “*”.

In group A, nine unique WG sequences were identified, with one sequence shared by 15 isolates and the other eight sequences distributed among 10 HAV isolates (Figure 2). In group B, one sequence was shared by eight isolates, another sequence by six isolates, and 20 isolates shared 18 different sequences. In Group C, the major sequence was shared by four isolates, and the second major sequence by three isolates, and yet another sequence by two isolates; the remaining eight different sequences were represented by single isolates. In Group D, two major sequences were shared by eight and three isolates, respectively, and the remaining 13 different sequences belonged to single isolates (Figure 2).

### Intra-host HAV diversity

**Figure 3:**
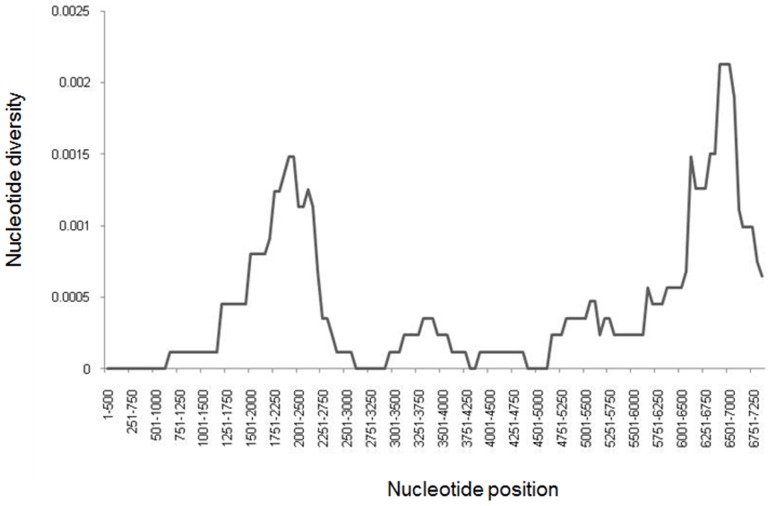
Nucleotide variability among strains of outbreak D. The graph was constructed using sliding window of 500 nt and steps of 250 nt.

Finding several HAV strains differing from each other at 6–23 nt sites in the onion- or oyster-related outbreaks suggests that the food items were likely contaminated with more than one HAV strain rather than single strains evolving in infected patients to produce the observed genetic heterogeneity seen in each outbreak. Thus, every infected patient was likely exposed to multiple viral variants, resulting in the establishment of a diverse intra-host viral population. To investigate whether each patient was infected with heterogeneous viral population, clonal analysis of HAV variants was conducted using EPLD. Scanning of the alignment of HAV sequences using a sliding window showed that the most variable region among the group B strains was located in the P3 region at nt positions 6000–7250 (Figure 3). This region was used to generate 30–40 clonal sequences for each patient. All sequences in each of the eight patients tested by EPLD were identical, suggesting that despite exposure to multiple viral variants, each tested patient became infected with a single HAV variant. Another possibility is that each patient was infected with few HAV variants but only one became dominant.

### Sporadic HAV strains

**Figure 4:**
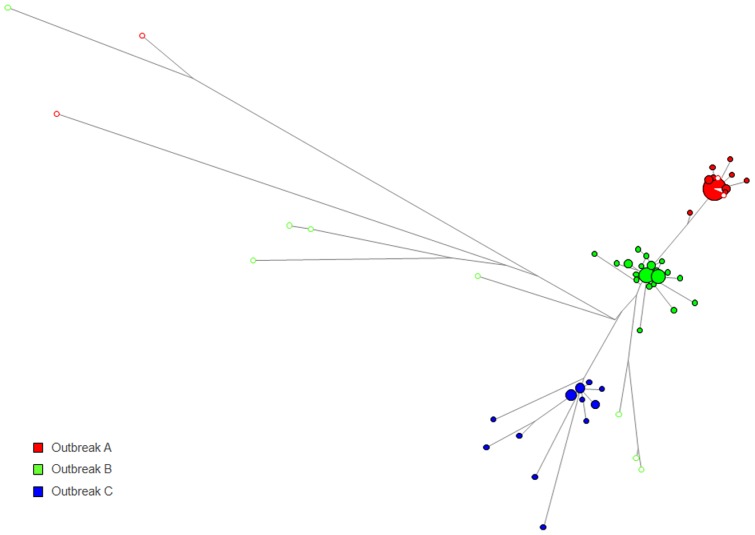
Genetic relationship between HAV strains associated with outbreaks A, B and C. Median joining network analysis was performed using strains from outbreaks A, B and C. Color code is as in Figure 1. Non-outbreak strains (open circles) are also color coded according to the outbreak with which they share the VP1/P2B sequences.

**Figure 5:**
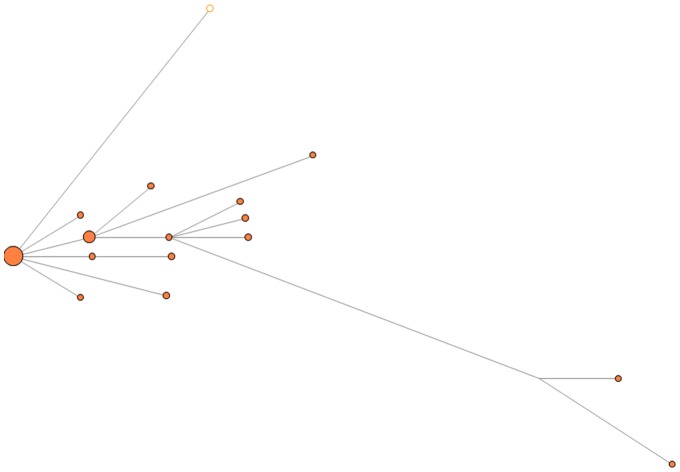
Genetic relationship between HAV strains associated with outbreak D. Median joining network analysis was performed using strains from outbreaks D. Color code is as in Figures 1 and 4. Non-outbreak strain sharing the same VP1/P2B sequences (open circle) is also depicted.

WG sequences were obtained from 14 HAV strains identified through the Sentinel Counties Surveillance for Acute Viral Hepatitis and Border Infectious Disease Surveillance Projects that shared identical VP1/P2B sequences with strains identified in the four outbreaks. Five strains were found to share the VP1/P2B sequence with group A, eight with group B, and one with group D. No isolates sharing the same VP1/P2B sequence with outbreak C were available. MJN analysis was used to visualize genetic relatedness among HAV isolates sharing the VP1/P2B sequences. Significant genetic differences were observed between the 10 sporadic strains and strains corresponding to groups A, B and D. The mean genetic distance was 0.64 (range, 0.42 to 0.88) (Figure 4). Three sporadic strains were genetically close to the group A strains, with a mean genetic distance of 0.02. A single sporadic strain sharing the VP1/P2B sequence with group D strains had a genetic distance of 0.08, indicating its close genetic association with the oyster-associated outbreak (Figure 5).

### Epidemiological association between sporadic and outbreak HAV strains

Review of surveillance data collected from sporadic case-patients revealed no direct epidemiologic associations with the foodborne outbreaks. Ten of the 14 sporadic cases had occurred either in geographic settings or calendar years distinct from those of the outbreak-related cases, or had been associated with foreign travel. Of the four remaining sporadic cases, however, there were temporal and geographic commonalities with outbreak-related cases. All four of these “sporadic” cases were genetically related to outbreak cases; three cases identified in 2003 from counties in Alabama and Washington were genetically related to strains from outbreaks A and B and the other case, identified in 2005 in Florida, was genetically related to outbreak D. Therefore, the temporal and geographic associations combined with genetic relatedness, suggest that these four cases, previously considered sporadic, were in fact likely outbreak-related.

### Evaluation of subgenomic regions

The observation of genetic heterogeneity among HAV isolates sharing the VP1/P2B sequence prompted a search for a genomic region that would be suitable for the accurate representation of phylogenetic relationships among the strains. Analysis of three genomic regions, 5′UTR, the 5′-end of VP1 and the entire VP1, which are commonly used for inferring phylogenetic relationships among HAV strains, showed that none of them accurately identified the HAV outbreak strains (Figure 1). Groups B and C were indistinguishable using 5′UTR (Fig. 1B), while variants from group A and the majority of group B variants were found clustered together using the 5′-end of VP1or the entire VP1(Fig. 1C, 1D and 1E). Some variants from group B were clustered separately from the majority of variants from this group when the VP1 regions were used for phylogenetic analysis (Fig. 1D and 1E).

**Figure 6:**
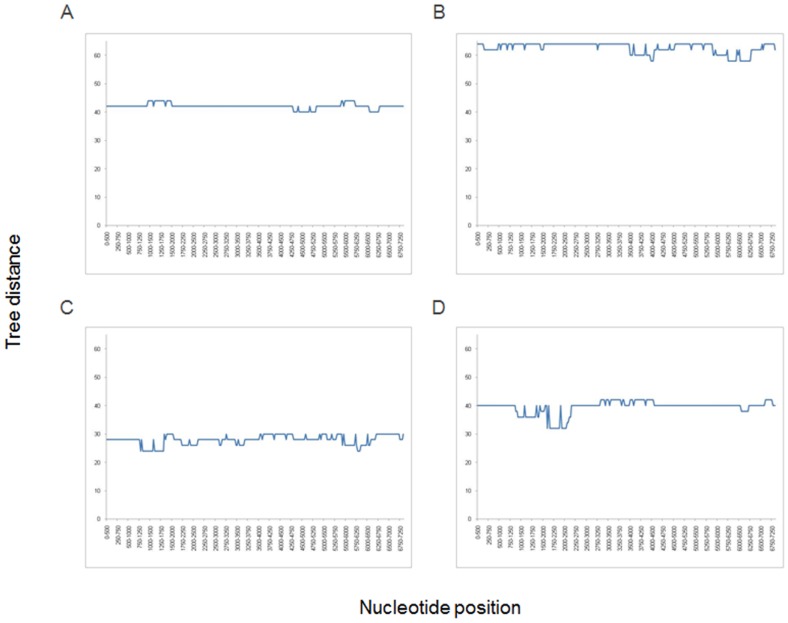
Tree topology analysis. Tree topology analysis using a sliding window (500 nt) approach. Trees constructed for each window were compared to the reference tree containing WG sequences from each outbreak.

**Figure 7:**
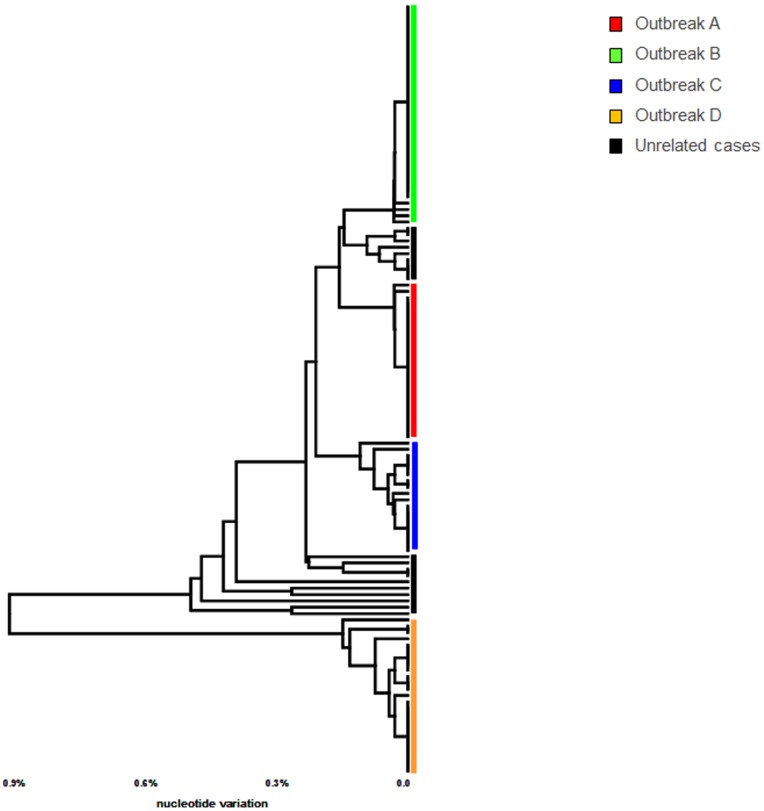
Phylogenetic analysis of VP1-P2C region from outbreak and sporadic HAV strains. Phylogenetic analysis of the HAV VP1-P2C region was performed using all HAV outbreak strains. Samples belonging to each outbreak are color coded.

Since none of the commonly used subgenomic regions represented phylogenetic relationships among HAV strains accurately, we conducted a WG scan. Phylogenetic trees were constructed for all 1370 windows and compared to the WG tree. Analysis showed that all windows produced trees equally inconsistent with the WG tree (Figure 6). However, analysis of a longer region at positions 2810–4510 (1700 nts long), encompassing the 3′-end of VP1 through most of P2C, resulted in a clear separation of the outbreak strains and the 14 sporadic strains (Figure 7). Nonetheless, phylogenetic trees constructed using P2C and WG did not match completely (data not shown).

## Discussion

Genetic analyses of WG sequences showed that HAV variants identified from food-borne outbreaks are heterogeneous, though closely related, despite sharing the VP1/P2B junction sequences. The degree of genetic diversity among HAV variants from a single outbreak varied, with HAV WG sequences being different at up to 23 nt sites among the onion and oyster-related outbreaks. The HAV sequence variants identified in each outbreak belonged to more than one strain. Although only one dominant strain was found in outbreak A, two to three dominant strains were identified in the other three outbreaks associated with consumption of green onions or oysters. Additionally, each outbreak was associated with many minority strains (Fig. 1).

The origin of such diversity among HAV variants in each outbreak is not known. It is conceivable that the observed genetic heterogeneity reflects diversity of viral strains circulating at the geographic location of the source of the implicated food items rather than the generation of viral variants from a single HAV ancestor during the course of infection of each host. The existence of more than one dominant strain in outbreaks B, C and D supports heterogeneous HAV sources rather than acquisition of identical substitutions by a single source-strain in groups of infected patient. However, it is possible that one of the dominant variant in each outbreak represents the ancestral strain; then, the other dominant variants in outbreaks B, C and D could have originated from the ancestral strain through extensive parallel evolution in several infected patients. Although, such frequent, independent acquisition of identical substitutions seems unlikely even under strong selection pressures. Alternatively, the observed genetic diversity could have been developed through a chain of transmissions. It is noteworthy that during outbreak C the contaminated onions were consumed in a single restaurant over a few days [17], which argues against the existence of widespread chains of transmission. Therefore, close genetic relatedness among HAV strains found in each outbreak are more likely to have resulted from viral evolution among many hosts in the locale where the contaminated food originated.

Consumption of food contaminated with a diverse HAV population may result in co-infection of each individual with more than one HAV strain. Detection of the intra-host HAV diversity reported earlier [26], [27] seemingly supports this suggestion. However, genetic analysis of intra-host HAV variants from individuals involved in the onion-related outbreak B showed a lack of detectable intra-host genetic diversity. Food items associated with the outbreaks studied here are usually washed during preparation, thus diluting the viral pool. Although HAV is tolerant to adverse environmental conditions for extended periods of time [22], [28], [29], it is approximately100-fold less infectious via peroral than intravenous inoculation as was shown in experimental infections of nonhuman primates [30]. Thus, the observed intra-host homogeneity can be explained by exposure of each individual to the limited number of viral particles, from which a single variant or few variants successfully established productive infection. The existence of HAV intra-host heterogeneity was not studied extensively in this study. However, recent publications have reported intra-host HAV heterogeneity in infected patients [26], [27] in contrast with our findings. Although this discrepancy can be attributed to differences in the employed experimental methodology for the detection of intra-host heterogeneity, it could also indicate variation in the number of HAV variants establishing infection in exposed individuals. Exposure to large inocula of HAV, from raw sewage for example, can be expected to lead to productive infection by a heterogeneous HAV population. By contrast, exposure to a small number of viral particles, as from contaminated food, would more likely result in productive infection by a homogeneous population. Thus, analysis of intra-host heterogeneity may provide important clues for characterization of the potential modes of HAV transmission. Further molecular investigations of HAV variants from outbreaks associated with different viral sources are warranted to uncover the association between the intra-host HAV diversity and modes of transmission.

Analysis of WG sequences offers a more complete genetic characterization of HAV strains than short subgenomic regions. In this study, HAV genetic identity was more precisely determined using WG sequences. Ten of 14 sporadic HAV strains that shared the VP1/P2B region with outbreak strains were clearly separated from these strains (Fig. 4). However, 4 sporadic strains were genetically close to strains from 2 outbreaks, suggesting their link to the outbreaks despite the lack of a clear epidemiological association between these sporadic and outbreak strains.

The molecular analysis showed that none of the 500 nt-regions accurately reflect genetic relatedness among HAV strains, suggesting a cautious use of subgenomic regions for the molecular tracking of transmissions. However, it should be noted that tracking does not require assessment of complete phylogenetic relationships and is plainly based on the identification of genetic proximity or identity. Additionally, the high prevalence of infection with genetically related HAV variants during the epidemiologically identified outbreaks significantly reduces the opportunity for false detection of transmissions, thus allowing for the reasonably efficient use of subgenomic regions. Identification of the sporadic and outbreak HAV strains sharing the VP1/P2B region indicates that tracking transmissions using subgenomic regions may be misleading when applied to HAV strains with unknown epidemiological association. These findings indicate that investigations should be conducted using HAV WG sequences.
